# Effect of Physical Activity and Dietary Changes on Management of Type 2 Diabetes Mellitus Patients: A Case–Control Study in Bangladesh

**DOI:** 10.1002/edm2.70051

**Published:** 2025-05-15

**Authors:** Hasan Mahmud Hadi, Md. Monir Hossain Shimul, Md. Sakhawath Hossain, Afroza Sultana, Md. Kamrul Hossain, Salamat Khandker, Salim Khan

**Affiliations:** ^1^ Department of Public Health Daffodil International University Dhaka Bangladesh; ^2^ Healthcare Pharmaceuticals Ltd. Dhaka Bangladesh; ^3^ Department of Business Administration Daffodil International University Dhaka Bangladesh; ^4^ Department of Computer Science and Engineering Daffodil International University Dhaka Bangladesh; ^5^ Chitkara University Institute of Engineering and Technology Chitkara University Punjab ‐ 140401 India; ^6^ Faculty of Health, Education and Life Sciences, Birmingham City University Birmingham UK

**Keywords:** Bangladesh, glycaemic control, lifestyle modifications, physical activity, type 2 diabetes mellitus (T2DM)

## Abstract

**Background:**

Type 2 diabetes mellitus (T2DM) is a growing public health issue in Bangladesh, projected to affect 13.7 million individuals by 2045. Physical inactivity and poor dietary habits increase the risk of complications. This study examined the effects of physical activity and lifestyle modifications on T2DM management.

**Methods:**

A case–control study was conducted at Manikganj District Diabetic Hospital with 334 T2DM patients (aged 45–60 years). The case group (*n* = 167) followed structured physical activity and dietary modifications, while the control group (*n* = 167) did not. Data on socio‐demographics, lifestyle and glycaemic markers—fasting plasma glucose (FPG), postprandial plasma glucose (PPG) and HbA1c—were collected at baseline, 3 months and 6 months. Chi‐square tests and ordinal logistic regression models assessed associations between lifestyle factors and glycaemic outcomes.

**Results:**

The control group had significantly higher plasma glucose levels, associated with gender (*p* < 0.001), treatment type (*p* = 0.004), medical adherence (*p* = 0.009), food habits (*p* = 0.007) and BMI (*p* = 0.005). The case group showed a significant FPG reduction from 10.035 mmol/L to 6.261 mmol/L (*p* < 0.001), with similar trends for PPG and HbA1c. Males had 1.8 times higher odds of elevated FPG, while poor adherence increased this risk 2.5‐fold.

**Conclusions:**

Lifestyle modifications significantly improve glycaemic control in T2DM patients. Strengthening adherence to medical advice and integrating structured interventions into routine care could enhance diabetes management in Bangladesh.

## Graphical abstract

Structured physical activity and dietary modifications significantly improved glycaemic control in T2DM patients in Bangladesh. The intervention group showed a marked reduction in fasting plasma glucose (10.0 → 6.3 mmol/L, *p* < 0.001), with better adherence leading to greater benefits. Lifestyle interventions should be integrated into routine diabetes management for improved outcomes.

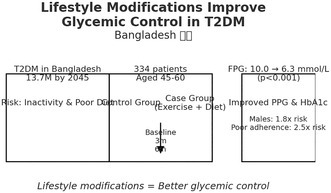

## 1 Introduction

Type 2 diabetes mellitus (T2DM) is a major global health concern with a rising prevalence, significantly impacting individuals and healthcare systems. In Bangladesh, the incidence of T2DM is increasing rapidly, projected to be 13.7 million by 2045 [1]. This chronic condition, characterised by insulin resistance and β‐cell dysfunction, is associated with severe complications such as cardiovascular disease, neuropathy and nephropathy [2]. Effective management of T2DM requires a comprehensive approach that combines pharmacological treatment with lifestyle modifications, notably physical activity and dietary changes [3].

Physical activity plays a crucial role in managing T2DM by enhancing insulin sensitivity, supporting weight management and improving cardiovascular health [4]. Even modest increases in physical activity have been shown to significantly improve glycaemic control and reduce the reliance on medications [5]. In Bangladesh, however, physical inactivity is prevalent among T2DM patients due to factors such as urbanisation, sedentary jobs and limited recreational facilities [6].

Lifestyle modifications, including diet, smoking cessation and alcohol reduction, are also vital for managing T2DM. A diet high in fibre and low in processed sugars improves glycaemic control and reduces diabetes‐related complications [7]. Smoking and excessive alcohol consumption further impair glycaemic control and increase cardiovascular risks [8]. In Bangladesh, dietary habits and lifestyle behaviours are shaped by cultural and socio‐economic factors, which may affect the success of general lifestyle recommendations [3].

The importance of investigating the effects of physical activity and lifestyle on T2DM patients in Bangladesh is significant for several reasons. First, understanding the relationships between physical activity, lifestyle and diabetes management is crucial for tailoring interventions to the specific needs of this population. Identifying lifestyle patterns that influence glycaemic control can facilitate the development of personalised treatment plans [9]. Second, such research provides insights that can guide preventive strategies to reduce T2DM incidence. By highlighting modifiable risk factors, public health initiatives can be designed to promote healthy behaviours and effectively address the diabetes burden in Bangladesh [10].

This study employs a case–control design to compare physical activity and lifestyle modifications between T2DM patients with varying glycaemic control. Despite evidence supporting lifestyle modifications for T2DM management, limited research exists on their application in low‐resource settings. This study fills that gap by identifying the effects of lifestyle interventions in T2DM management and provides actionable insights to healthcare providers, enabling more targeted strategies to mitigate the T2DM burden in Bangladesh.

## 2 Methodology

### 2.1 Study Design and Settings

This case–control study was conducted at the Manikganj District Diabetic Hospital which is situated in central Bangladesh and is part of Dhaka Division. The hospital serves the needs of male and female patients aged 45 to 60 years. The study aimed to investigate the effect of physical activity and lifestyle on the management of type 2 diabetes mellitus (T2DM) through an interventional design that assessed structured lifestyle modifications.

### 2.2 Sample Size Calculation and Sampling

The Sample size was calculated using the formula $n=\frac{z^{2}\mathrm{pq}}{d^{2}}$. In terms of the prevalence of diabetes in Bangladesh according to Talukder and Hossain (2022) [11], the *p* value was 0.32 and the calculated sample size was 334. This pairing inherently enhances statistical power and precision, which justifies the use of a prevalence‐based formula for the total sample size, further supported by GPower software validation that confirmed 334 participants were sufficient to detect medium effect sizes (80% power and 5% significance). This study employed an interventional design to compare glycaemic outcomes between a case group receiving structured interventions and a control group without structured interventions. A total of 334 type 2 diabetes patients aged between 45 and 60 years were selected using systematic random sampling from a hospital registry. Data collection occurred over 6 months, ensuring comprehensive needed follow‐up. A total of 167 patients (case group) were prescribed regular exercise in addition to a healthy diet as per the patients clinicians advice. A further 167 patients (control group) were selected where age, sex and socioeconomic conditions were matched to the case group. The control group patients were selected on the basis that they had made evident they were reluctant to exercise regularly or maintain a healthy diet as per previous clinician's advice. Cases were identified as T2DM patients participating in structured interventions, while controls were patients receiving usual care. Participants were matched on age (±5 years), income and education levels. A blinded selection process ensured objectivity.

### 2.3 Questionnaire

A comprehensive questionnaire was developed for data collection. Initially drafted in English, the questionnaire was translated into Bengali (the national language of Bangladesh) and then further re‐translated back into English to ensure cultural relevance. The data collection tool was pre‐tested and refined through several rounds of revisions to ensure it was user‐friendly and aligned with the study objectives. The final version was used for data collection during face‐to‐face interviews conducted by the principal investigator. Immediate on‐site scrutiny of the completed questionnaires was performed to identify and correct any discrepancies or errors, ensuring data accuracy.

### 2.4 Data Collection and Variables

A systematic process was employed by the principal investigator who conducted interviews with each patient to complete the requisite sections of the questionnaire. Standard procedures were followed to measure body weight and height—used to calculate body mass index (BMI); blood pressure and additional information relating to socio‐demographic factors, duration of dietary practices, type of treatment, comorbidities and physical exercise history. Control group participants continued their usual care as prescribed by their clinicians. The case group received structured interventions as part of the study design, which adhered to ethical standards.

Additionally, laboratory investigations were conducted to measure fasting plasma glucose (FPG), postprandial plasma glucose, HbA1c%, haemoglobin levels, blood cholesterol and serum creatinine. Physical activity was assessed using a validated questionnaire, while dietary intake was recorded via 24‐h recall and glycaemic parameters (FPG, PPG and HbA1c) were measured using standard clinical assays at baseline, 3 months and 6 months to assess changes over time. Volunteers in the case group regularly monitored adherence to prescribed lifestyle modifications, with follow‐ups conducted to evaluate the intervention's efficacy.

### 2.5 Operational Definitions and Quality Control

Regular exercise was defined as engaging in at least 150 min of moderate‐intensity activity per week, verified through weekly logs and volunteer‐conducted interviews. A healthy diet was characterised by balanced meals adhering to the Bangladeshi Dietary Guidelines, including low sugar, high fibre and reduced saturated fat intake. Lifestyle encompassed adherence to both exercise and diet, assessed through biweekly interviews and checklist reviews. Reluctance to exercise was defined as self‐reported non‐compliance with exercise guidelines or refusal to participate in structured activities despite medical advice. Volunteers ensured adherence in the case group by conducting weekly follow‐ups, maintaining logs and verifying self‐reports during follow‐up visits.

### 2.6 Data Analysis

The collected dataset was coded, cleaned and analysed using SPSS version 25 and Microsoft Excel 2019. Descriptive statistics were used to summarise the data and inferential statistics (chi‐square test) were employed to examine associations between variables. A proportional odds model was used to predict FPG, PPG and HbA1c based on the entire dataset, and its performance was assessed through test datasets.

### 2.7 Ethical Clearance

The study received ethical approval from the Research Ethics Committee of the Faculty of Health and Life Sciences, Daffodil International University, Dhaka, Bangladesh (Approval Reference: FAHSREC/DIU/2023/SMIG‐11). Additional permission for data collection was granted by the hospital director at Manikganj District Diabetic Hospital (Approval Reference: MDDH/2023/REC/002). The study adhered to all ethical guidelines and institutional policies.

### 2.8 Human Ethics and Consent to Participate

All participants were informed about the purpose, methods, potential risks and benefits of the study through a participant information sheet provided in Bengali and English. Written informed consent was obtained from each participant before data collection commenced. Participants were assured of the confidentiality and anonymity of their responses and informed of their right to withdraw from the study at any time without consequences. Blood investigations (e.g., FPG, PPG, and HbA1c) were part of routine clinical care and patients had covered the costs. All investigations were conducted in the study hospital and informed consent was also obtained for the inclusion of these parameters in research analysis. The study was conducted in compliance with the ethical principles of the Declaration of Helsinki and no vulnerable groups or forms of coercion were involved in the consent process.

## 3 Results

A similar gender distribution was evident between the case and control group. The majority of participants in both groups have education levels of ‘Secondary and below’, with a lower level seen in the control group. Additionally, more of the control group participants had a professional background although a greater percentage of participants were job holders in the case group. Both groups predominantly reside in semi‐furnished houses and maintain joint family structures, indicating similar socioeconomic environments. A high percentage of participants in both groups adhere to medical advice (91.0% and 92.8% control and case group, respectively) following dietary recommendations, however, the case group demonstrated higher adherence and additional structured interventions, contributing to distinct outcomes (Table 1).

**TABLE 1:** Socio‐demographic characteristics of participants.

Variables	Control group (*n* = 167)		Case group (*n* = 167)	
	Frequency	Percent	Frequency	Percent
Gender				
Male	91	54.5	95	56.9
Female	76	45.5	72	43.1
Level of education				
None	01	0.6	07	4.2
Secondary and below	102	61.1	81	48.5
Higher secondary to graduation	61	36.5	74	44.3
Postgraduate and above	03	1.8	05	3.0
Profession				
Farmer	09	5.4	07	4.2
Driver	01	0.6	04	2.4
Business	54	32.3	44	26.3
Banker	00	0.0	01	0.6
Job holder	20	12.0	39	23.4
Teacher	12	7.2	06	3.6
House wife	71	42.5	66	39.5
Housing condition of the patients				
Furnished (Pacca)	23	13.8	22	13.2
Semi‐furnished (Pacca)	109	65.3	110	65.9
Mud house	35	20.9	35	20.9
Family type				
Joint	93	55.7	93	55.7
Nuclear	74	44.3	74	44.3
Advised to maintain diet				
Yes	152	91.0	155	92.8
No	15	9.0	12	7.2
Doctors advise				
Only taking medicine	112	67.1	10	6.0
Only taking regular exercise	30	18.0	12	7.0.2
Taking medicine and regular exercise	25	15.0	145	86.8
Income category				
15,000 or below	24	14.4	14	8.4
15,001 to 30,000	123	73.7	143	85.6
Above 30,000	20	12.0	10	6.0
Total	167	100.0	167	100.0

The control group includes around 46% of participants with diabetes for more than 5 years, while around 52% of the case group has had diabetes for 3–5 years. Both groups primarily manage diabetes through a combination of insulin and oral antidiabetic treatments. Dietary choices vary, with a common caloric intake of 1400 Kcal/Day, though many participants do not strictly maintain their dietary habits. Notably, 95.2% of the Control Group and 95.8% of the Case Group have not undergone surgery following their diabetes diagnosis. However, surgeries such as eye surgery and open‐heart surgery were reported among participants before their diabetes diagnosis (Table 2).

**TABLE 2:** Distribution of respondents according to DM duration, treatment and Diet type.

Variables	Control group (*n* = 167)		Case group (*n* = 167)	
	Frequency	Percent	Frequency	Percent
Duration of DM				
Less than 3 years	27	16.2	31	18.6
3–5 years	64	38.3	66	39.5
Greater than 5 years	76	45.5	70	41.9
Type of treatment				
Only insulin	01	0.6	00	00
Only OAD	43	25.7	27	16.2
Insulin and OAD	123	73.7	140	83.8
Diet type				
No diet	17	10.2	12	7.2
1400 Kcal/day	49	29.3	63	37.7
1500 Kcal/day	20	12.0	37	22.2
1800 Kcal/day	29	17.4	18	10.8
1900 Kcal/day	46	27.5	34	20.4
2000 Kcal/day	06	3.6	03	1.8
Strictly maintained food habits				
Yes	55	32.9	90	53.9
No	122	67.1	77	46.1
Undergone any surgery				
Yes	08	4.8	7	4.2
No	159	95.2	160	95.8
Total	167	100.0	167	100.0

Exercise patterns reveal that 17.4% of the control group engages in regular exercise, compared to only 6.0% of the case group, with walking being the most common form (Table 3).

**TABLE 3:** Distribution of respondents according to exercise habits and types.

	Control group (*n* = 167)		Case group (*n* = 167)	
	Frequency	Percent	Frequency	Percent
Do exercise				
Yes	29	17.4	167	100
No	138	82.6	00	0.0
Exercise type				
No exercise (N/A)	138	82.6	00	0.0
Walking	24	14.4	87	52.1
Agricultural work	01	0.6	32	19.2
Freehand	01	0.6	24	14.4
Walking and freehand	02	1.2	17	10.2
Walking and cycling	01	0.6	07	4.2
Do exercise regularly				
Yes	10	6.0	133	79.6
No	157	94.01	34	20.4
Total	167	100.0	167	100.0

Both groups present with various co‐morbidities, with hypertension being the most prevalent followed by joint pain. Smoking and alcohol consumption are relatively uncommon in both groups. Body Mass Index (BMI) distribution shows that 48.5% of the control group falls within the healthy BMI range, 38.3% categorised as overweight and 13.2% as obese. In comparison, t 45.5% are in the healthy BMI range, 30.5% are categorised as overweight and 24.0% are categorised as obese in the case group. Income analysis reveals that the case group had greater numbers of participants within the medium income range (Table 4).

**TABLE 4:** Distribution of respondents according to co‐morbidities, BMI, monthly income and smoking or alcohol consumption habit (*n* = 167).

	Control group (*n* = 167)		Case group (*n* = 167)	
	Frequency	Percent	Frequency	Percent
Co‐morbidity (*n* = 167)				
Yes	73	43.7	73	43.7
No	94	56.3	94	56.3
Comorbidity types* (*n* = 73) multiple response				
Hypertension	55	32.9	45	26.9
Hypertension with IHD	9	5.4	11	6.6
BHP	04	2.4	04	2.4
Joint pain	43	25.7	51	30.5
UTI	02	1.2	07	4.2
Skin disease	01	0.6	03	1.8
Kidney problem	01	0.6	01	0.6
Stroke	02	1.2	01	0.6
Liver problem	01	0.6	01	0.6
Smoking, drinking alcohol (*n* = 167)				
Yes	34	20.4	28	16.8
No	133	79.6	139	83.2
BMI category				
18.5 < BMI < 25	81	48.5	76	45.5
25 < BMI < 30	64	38.3	51	30.5
BMI > 30	22	13.2	40	24.0
Total	167	100.0	167	100.0

*Note:* * indicates Multiple responses.

The mean fasting plasma glucose (FPG) in the control group showed an increasing trend, ranging from 8.69 to 9.99 mmol/L across follow‐ups, while postprandial plasma glucose (PPG) ranged from 12.52 to 15.93 mmol/L. Haemoglobin A1C (HbA1c) levels fluctuated between 8.75% and 9.758%. In contrast, the case group showed a significant decreasing trend across the three follow‐ups. The mean FPG in the case group dropped from 10.04 mmol/L in the first follow‐up to 6.261 mmol/L in the third follow‐up, with corresponding standard deviations of 2.2, 1.0 and 0.4, respectively (*p* < 0.01). Similarly, PPG levels decreased from 16.33 mmol/L (SD = 3.6) in the first follow‐up to 8.341 mmol/L (SD = 0.8) in the third follow‐up (*p* < 0.01). HbA1c% in the case group also followed a decreasing pattern, falling from 9.86% (SD = 1.7) to 6.62% (SD = 0.8) by the third follow‐up (*p* < 0.01). Haemoglobin levels showed a slight increase in both groups over time, but with no statistically significant difference in the control group, while the case group demonstrated a significant increase (*p* < 0.01).

**FIGURE 1:**
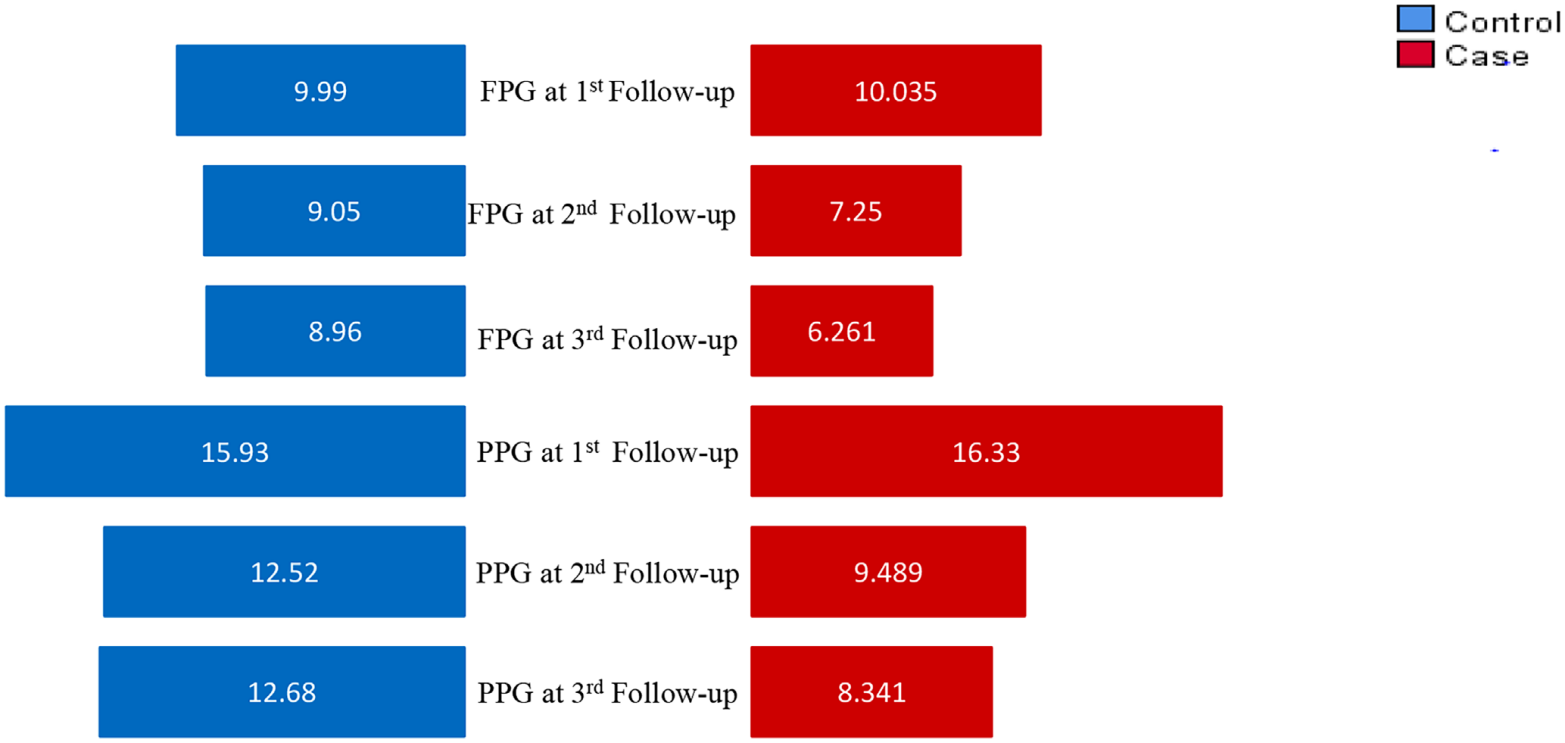
Comparison plot for mean FPG and PPG in different follow‐up.

Lipid profiles in both groups exhibited varying trends. In the case group, triglycerides, LDL and total cholesterol showed a consistent decrease, while HDL increased significantly (*p* = 0.004). For triglycerides, the mean dropped from 207.45 mmol/L (SD = 45.3) to 169.01 mmol/L (SD = 25.6) (*p* < 0.01) and LDL levels decreased from 100.13 mmol/L (SD = 30.8) to 76.468 mmol/L (SD = 19.9) (*p* = 0.009). In contrast, HDL levels increased from 39.63 mmol/L to 49.407 mmol/L (*p* = 0.004). For kidney function (serum creatinine) and liver enzyme levels (SGPT), there were no significant changes in serum creatinine for either group, while SGPT showed a slight but statistically significant reduction in the case group (*p* = 0.038). These results highlight the effectiveness of the interventions in the case group, particularly in glucose regulation and lipid profile improvement (Table 5, Figure 1).

**TABLE 5:** Laboratory parameters of the control and case group respondents during follow‐up visits.

Parameters	First follow‐up Mean±	Range (Min, Max)	Second follow‐up Mean	Range (Min, Max)	Third follow‐up Mean	Range (Min, Max)	*p*
Laboratory parameter of control group							
FPG	9.99 ± 2.08	11.25 (5.50,16.75)	9.034 ± 1.6	13.48 (6.20,19.68)	8.69 ± 9.28	9.28 (6.5,15.78)	0.156
PPG	15.93 ± 3.33	18.76 (6.32,25.08)	12.52 ± 1.7	10.53 (6.50,17.03)	12.68 ± 8.3	8.3 (9.1,17.4)	< 0.01
HbA1C %	9.758 ± 1.51	7.44 (7.36,14.80)	8.94 ± 1.15	4.97 (7.01,11.98)	8.75 ± 4.66	4.66 (6.9,11.56)	< 0.01
Hbg level	12.77 ± 1.25	5.5 (9.10,14.6)	13.07 ± 1.06	4.36 (10.2,14.56)	13.23 ± 4.16	4.16 (10.45,14.6)	0.160
Sr. triglycerides	219.52 ± 77.56	674 (−106,780)	209.7 ± 65.39	598 (−92,690)	290.09 ± 0	492 (−88,580)	< 0.01
Sr. LDL	108.85 ± 35.64	177 (−42,219)	104.9 ± 30.52	165 (−40,205)	103.99 ± 0	163 (−39,202)	0.220
Sr. HDL	41.18 ± 6.97	43 (19,62)	40.79 ± 6.14	39 (21,60)	39.37 ± 0	28 (26,54)	0.002
Total cholesterol	184.76 ± 27.11	175 (−106,281)	183.2 ± 24.18	155 (−121,276)	185 ± 0	209 (−56,265)	0.837
Sr. creatinine	0.8891 ± 0.14	0.62 (0.60,1.22) 71 (14,85)	0.879 ± 16.12	0.57 (0.62,1.19) 60 (18,78)	0.89 ± 0	0.59 (0.62,1.21) 53 (19,72)	0.381
SGPT	31.79 ± 12.47	71 (14,85)	32.8 ± 11.14	60 (18,78)	34.92 ± 0	53 (19,72)	0.137
Laboratory parameter of case group							
FPG	10.04 ± 2.2	11.25 (5.50,16.75)	7.25 ± 1	13.48 (6.20,19.68)	6.261 ± 0.4	9.28 (6.5,15.78)	< 0.01
PPG	16.33 ± 3.6	18.76 (6.32,25.08)	9.489 ± 1.5	10.53 (6.50,17.03)	8.341 ± 0.8	8.3 (9.1,17.4)	< 0.01
HbA1C %	9.86 ± 1.7	7.44 (7.36,14.80)	7.746 ± 1.2	4.97 (7.01,11.98)	6.62 ± 0.8	4.66 (6.9,11.56)	< 0.01
hbg level	13.08 ± 1.3	5.5 (9.10,14.6)	13.23 ± 1	4.36 (10.2,14.56)	13.45 ± 0.9	4.16 (10.45,14.6)	< 0.01
Sr. triglycerides	207.45 ± 45.3	674 (−106,780)	183.6 ± 32	598 (−92,690)	169.01 ± 25.6	492 (−88,580)	< 0.01
Sr. LDL	100.13 ± 30.8	177 (−42,219)	87.25 ± 24.1	165 (−40,205)	76.468 ± 19.9	163 (−39,202)	0.009
Sr. HDL	39.63 ± 6.3	43 (19,62)	43.59 ± 4.7	39 (21,60)	49.407 ± 5.3	28 (26,54)	0.004
Total cholesterol	177.45 ± 27.5	175 (−106,281)	166.8 ± 22.2	155 (−121,276)	161.99 ± 20.2	209 (−56,265)	0.676
Sr. creatinine	0.86 ± 0.1	0.62 (0.60,1.22)	0.852 ± 0.1	0.57 (0.62,1.19)	0.842 ± 0.1	0.59 (0.62,1.21)	0.474
SGPT	27.42 ± 8	71 (14,85)	26.3 ± 6.7	60 (18,78)	25.64 ± 7.9	53 (19,72)	0.038

*Note:* *p* Value from ANOVA.

Figure 1 illustrates a notable decrease in both FPG and PPG levels in the case group compared to the control group. This visual representation shows more favourable outcomes in glycaemic management for the case group.

Table 6 highlights that the case group exhibits a positive trend, with an increase in the number of respondents with normal FPG levels and a simultaneous decline in elevated PPG and HbA1c levels over successive follow‐ups. In contrast, the control group maintains relatively stable glycemic levels, predominantly within the normal range for both FPG and PPG. The case group also shows significant improvements in lipid management, including a reduction in the high‐risk category for triglycerides from 53.3% to 5.3% and a significant achievement of desirable LDL levels (99.4%) by the third follow‐up. HDL levels improved significantly in the case group, from 15.0% to 78.4%. Additionally, there is a reduction in the high‐risk category for total cholesterol from 3.0% to 0%. Serum creatinine levels, predominantly within the normal range, show consistent improvement over time in the case group. SGPT levels also shift favourably towards normalcy, with a substantial increase in the normal category for the case group. Haemoglobin levels exhibit a positive pattern, with a notable decrease in anaemic cases and an overall improvement in haemoglobin status across follow‐ups for both groups.

**TABLE 6:** Frequency distribution of fasting and postprandial plasma glucose, HbA1C% and lipid profile.

Fasting, postprandial plasma glucose, lipid profile in mmol/L and HbA1C%							
Variable	Level	First follow‐up		Second follow‐up		Third follow‐up	
		Control	Case	Control	Case	Control	Case
		Frequency (Percent)	Frequency (Percent)	Frequency (Percent)	Frequency (Percent)	Frequency (Percent)	Frequency (Percent)
Fasting plasma glucose, Post prandial plasma glucose and HbA1C							
FPG	< 8	23 (13.8)	25 (15)	44 (26.3)	139 (83.2)	56 (33.5)	167 (100)
	8–12	118 (70.7)	110 (65.9)	117 (70.1)	27 (16.2)	110 (65.9)	0.0 (0)
	> 12	26 (15.6)	32 (19.2)	06 (3.6)	01 (0.6)	01 (0.6)	00 (0.0)
	*p* Value^a^	0.611		< 0.01		< 0.01	
PPG	12–14	36 (0.6)	39 (23.4)	70 (41.9)	39 (23.4)	92 (55.1)	51 (30.5)
	14.1–20	112 (7.8)	102 (61.1)	97 (58.1)	128 (76.6)	75 (45.9)	116 (69.5)
	> 20	19 (91.6)	26 (15.6)	00 (0.0)	00 (0.0)	00 (0.0)	00 (0.0)
	*p* Value^a^	0.433		0.002		< 0.01	
HbA1C.	< 6.5	00 (0.0)	00 (0.0)	00 (0.0)	07 (4.2)	00 (0.0)	90 (53.9)
	6.5–10	106 (63.5)	104 (62.3)	136 (81.4)	146 (87.4)	141 (84.4)	77 (46.1)
	High (> 10)	61 (36.5)	63 (37.7)	31 (18.6)	14 (8.4)	26 (15.6)	00 (0.0)
	*p* Value^a^	0.975		0.001		< 0.001	
Lipid profile							
Sr. triglycerides	* < 150	17 (10.2)	17 (10.2)	18 (10.8)	24 (14.4)	18 (10.8)	35 (21.0)
	**150–200	61 (36.5)	61 (36.5)	70 (41.9)	92 (55.1)	73 (43.7)	123 (73.7)
	*** > 200	89 (53.3)	89 (53.3)	79 (47.3)	51 (30.5)	76 (45.5)	09 (5.3)
	*p* Value^a^	0.999		0.007		< 0.001	
Sr. LDL. categories	* < 130	123 (73.7)	135 (80.8)	135 (80.8)	155 (92.8)	137 (82.0)	166 (99.4)
	**130–160	24 (14.4)	22 (13.2)	24 (14.4)	12 (7.2)	26 (15.6)	01 (0.6)
	***> 160	20 (12.0)	10 (6.0)	08 (4.8)	00 (0.0)	04 (2.4)	00 (0.0)
	*p* Value^a^	0.137		0.001		< 0.001	
Sr. HDL. categories	* > 45	39 (23.4)	25 (15.0)	39 (23.4)	57 (34.1)	27 (16.2)	131 (78.4)
	**35–45	102 (61.1)	100 (59.9)	107 (64.0)	108 (64.7)	104 (62.3)	36 (21.6)
	*** < 35	26 (15.6)	42 (25.1)	21 (12.6)	02 (1.2)	36 (21.6)	00 (0.0)
	*p* Value^a^	0.033		< 0.001		< 0.001	
Total cholesterol	* < 200	127 (76.0)	140 (83.8)	135 (80.8)	160 (95.8)	128 (76.6)	162 (97.0)
	**200–240	35 (21.0)	22 (13.2)	28 (16.8)	06 (3.6)	34 (20.4)	05 (3.0)
	*** > 240	05 (3.0)	05 (3.0)	04 (2.4)	01 (0.6)	05 (3.0)	00 (0.0)
	*p* Value^a^	0.165		< 0.001		< 0.001	
Sr. creatinine, SGPT, haemoglobin							
Sr. creatinine	0.5–1.0	143 (85.6)	147 (88.0)	140 (83.8)	155 (92.8)	133 (80.2)	159 (95.2)
	Above 1.0	24 (14.4)	20 (12.0)	27 (16.2)	12 (7.2)	33 (19.8)	8 (4.8)
	*p* Value^a^	0.627		0.017		< 0.001	
SGPT categories	Below 35	122 (73.1)	138 (82.6)	114 (68.3)	143 (85.6)	100 (59.9)	150 (89.8)
	Above 35	45 (26.9)	29 (17.4)	53 (31.7)	24 (14.4)	67 (40.1)	17 (10.2)
	*p* Value^a^	0.048		< 0.001		< 0.001	
Haemoglobin categories	< 11	13 (7.8)	4 (2.4)	7 (4.2)	1 (0.6)	5 (3.0)	0 (0)
	11–13	66 (39.5)	69 (41.3)	60 (35.9)	66 (39.5)	54 (32.3)	58 (34.7)
	Above 13	88 (52.7)	94 (56.3)	100 (59.9)	100 (59.9)	108 (64.7)	109 (65.3)
	*p* Value^a^	0.081		0.091		0.076	
	Total	167 (100)	167 (100)	167 (100)	167 (100)	167 (100)	167 (100)

*Note:* *, ** and *** indicate desirable, borderline and high‐risk respectively.

^**a**^ Indicates the *p*‐value calculated for the association between the case and control groups of the same follow‐up using the Chi‐Square test.

**FIGURE 2:**
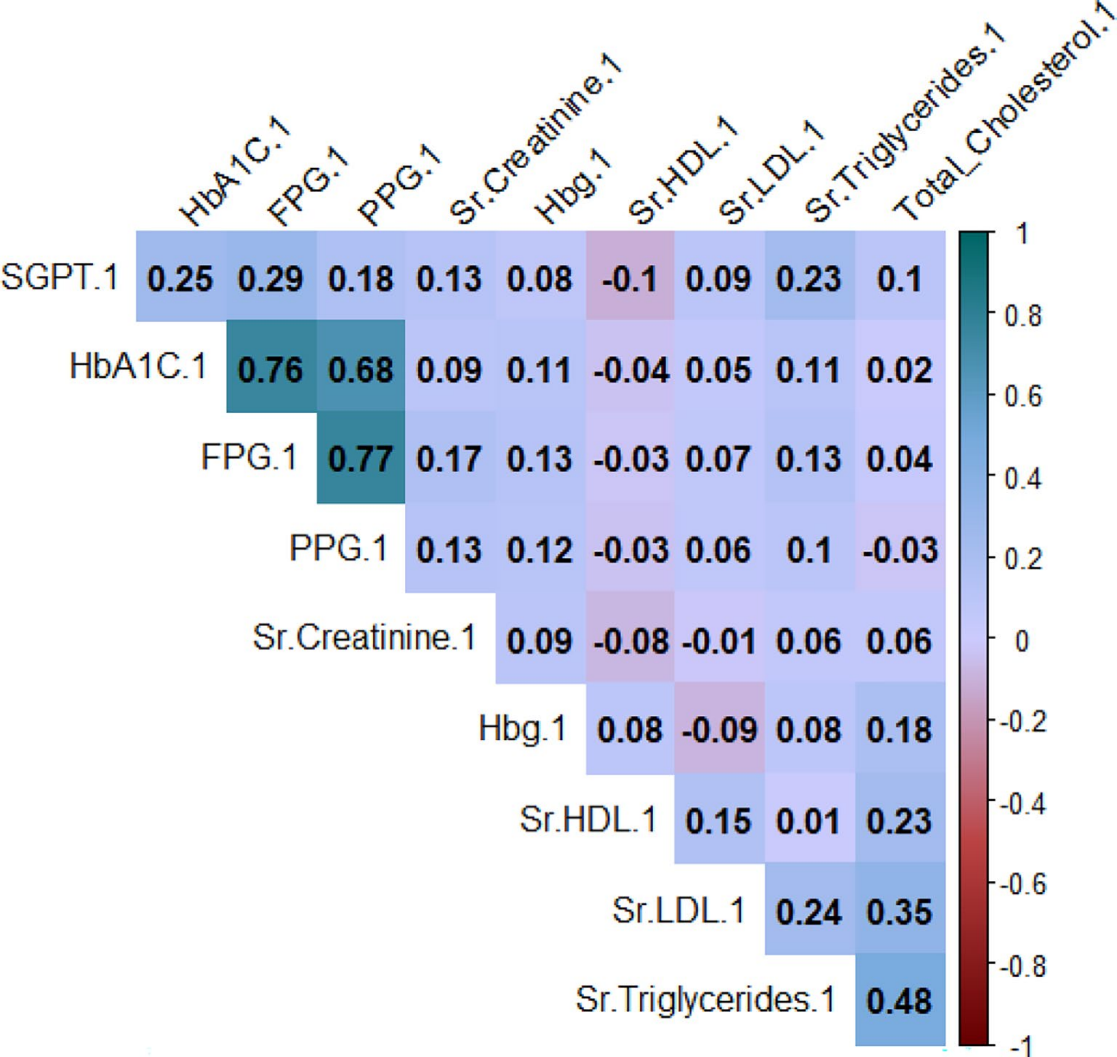
Correlation matrix of diagnosis parameters at first follow‐up.

**FIGURE 3:**
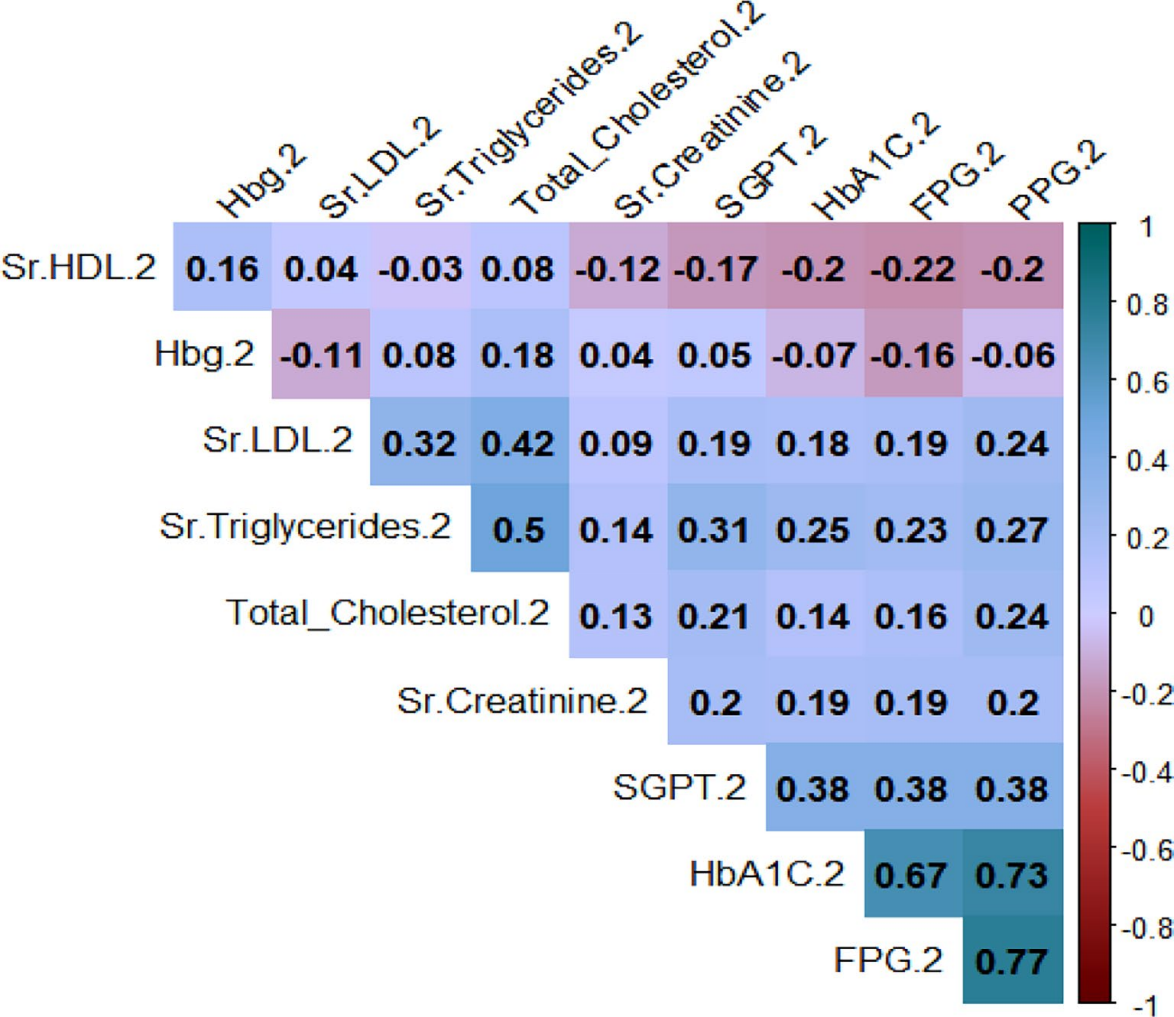
Correlation matrix of diagnosis parameters at second follow‐up.

**FIGURE 4:**
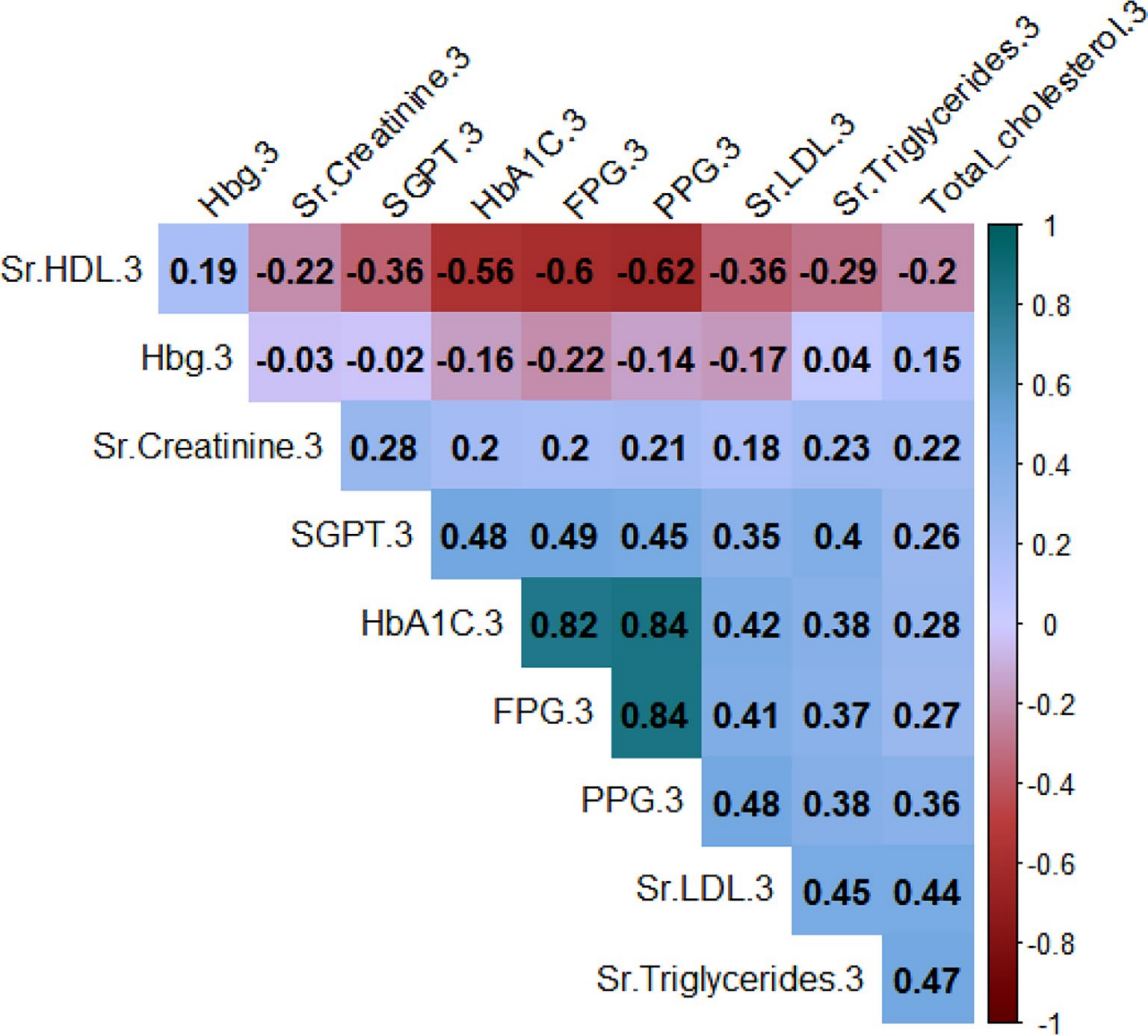
Correlation matrix of diagnosis parameters at third follow‐up.

Figures 2, 3, 4 reveal strong positive correlations between fasting plasma glucose (FPG) and postprandial glucose (PPG), with coefficients ranging from 0.77 to 0.84, indicating consistent associations between these measures. Glycated haemoglobin (HbA1C) also shows substantial positive correlations with both FPG and PPG, with values between 0.73 and 0.82, emphasising the relationship between HbA1C and glucose levels. Additionally, a moderate positive correlation (0.25) exists between serum triglycerides and HbA1C, while a weak negative correlation (−0.07) is observed between HbA1C and haemoglobin.

Table 7 highlights that gender (*p* = 0.000), treatment type (*p* = 0.004), adherence to medical advice (*p* = 0.009), food habits (*p* = 0.007) and BMI (*p* = 0.005) are significantly associated with elevated plasma glucose levels. During the second follow‐up, males continued to exhibit higher glucose levels compared to females (*p* = 0.016), with adherence to medical advice showing a highly significant impact on glycaemic control (*p* < 0.001). However, other factors like food habits and BMI did not show significant associations in this follow‐up. By the third follow‐up, gender remained significantly associated with glucose levels (*p* = 0.003), with males consistently displaying higher rates. Adherence to medical advice continued to be a critical factor (*p* < 0.001), while the significance of food habits, exercise and BMI varied, indicating that their impact on glucose levels may fluctuate over time.

**TABLE 7:** Association measure of FPG at first, second and third follow‐ups with other relevant variables.

Name of variables	< 8 mmol/L	8 to 12 mmL/L	> 12 mmol/L	Chi‐square (*p*)
Fasting plasma glucose level at first follow‐up				
Gender				
Male	15 (31.3)	130 (57)	41 (70.7)	
Female	33 (68.8)	98 (43)	17 (29.3)	
Treatment type				15.524 (0.004)
Only insulin	0 (0)	1 (0.4)	0 (0)	
Only OAD	20 (41.7)	42 (18.4)	8 (13.8)	
Insulin and OAD	28 (58.3)	185 (81.1)	50 (86.2)	
Follow doctors' advice				13.408 (0.009)
Take medicine	22 (45.8)	77 (33.8)	23 (39.7)	
Regular exercise	8 (16.7)	34 (14.9)	0 (0)	
Take medicine and regular exercise	18 (37.5)	117 (51.3)	35 (60.3)	
Maintained food habit				9.793 (0.007)
Yes	21 (43.8)	59 (25.9)	10 (17.2)	
No	27 (56.3)	169 (74.1)	48 (82.8)	
BMI				15.032 (0.005)
18.5 < BMI < 25	14 (29.2)	113 (49.6)	30 (51.7)	
25 < BMI < 30	17 (35.4)	75 (32.9)	23 (39.7)	
BMI > 30	17 (35.4)	40 (17.5)	5 (8.6)	
Fasting plasma glucose at second follow up visit				
Gender				8.246 (0.016)
Male	114 (237.5)	70 (30.7)	2 (3.4)	
Female	69 (143.8)	74 (32.5)	5 (8.6)	
Treatment type				1.187 (0.880)
Only insulin	1 (2.1)	0 (0)	0 (0)	
Only OAD	40 (83.3)	29 (12.7)	1 (1.7)	
Insulin and OAD	142 (295.8)	115 (50.4)	6 (10.3)	
Follow doctors' advice				42.918 (0.000)
Take medicine	44 (91.7)	73 (32)	5 (8.6)	
Regular exercise	17 (35.4)	25 (11)	0 (0)	
Take medicine and regular exercise	122 (254.2)	46 (20.2)	2 (3.4)	
Maintained food habit				3.843 (0.146)
Yes	57 (118.8)	32 (14)	1 (1.7)	
No	126 (262.5)	112 (49.1)	6 (10.3)	
BMI				6.701 (0.153)
18.5 < BMI < 25	92 (191.7)	61 (26.8)	4 (6.9)	
25 < BMI < 30	54 (112.5)	60 (26.3)	1 (1.7)	
BMI > 30	37 (77.1)	23 (10.1)	2 (3.4)	
Fasting plasma glucose level at third follow up‐visit				
Gender				11.752 (0.003)
Male	138 (287.5)	47 (20.6)	1 (1.7)	
Female	85 (177.1)	63 (27.6)	0 (0)	
Treatment type				7.373 (0.117)
Only insulin	0 (0)	1 (0.4)	0 (0)	
Only OAD	42 (87.5)	27 (11.8)	1 (1.7)	
Insulin and OAD	181 (377.1)	82 (36)	0 (0)	
Follow doctors' advice				85.549 (0.000)
Take medicine	50 (104.2)	71 (31.1)	1 (1.7)	
Regular exercise	20 (41.7)	22 (9.6)	0 (0)	
Take medicine and regular exercise	153 (318.8)	17 (7.5)	0 (0)	
Maintained food habit				5.543 (0.063)
Yes	66 (137.5)	23 (10.1)	1 (1.7)	
No	157 (327.1)	87 (38.2)	0 (0)	
BMI				5.490 (0.241)
18.5 < BMI < 25	108 (225)	6 (2.6)	1 (1.7)	
25 < BMI < 30	69 (143.8)	16 (7)	0 (0)	
> 30	46 (95.8)	110 (48.2)	0 (0)	

Gender (*p* = 0.697) and treatment type (*p* = 0.657) show no significant association with average Fasting Plasma Glucose (FPG) levels, suggesting similar distributions between males and females. However, adherence to medical advice significantly impacts FPG levels (*p* < 0.001), underscoring the importance of medical guidance in diabetes management. Additionally, a sedentary lifestyle (*p* = 0.006) is significantly associated with higher FPG levels, highlighting the critical role of physical activity, while BMI shows a borderline association (*p* = 0.091), suggesting a potential influence on FPG levels (Table 8).

**TABLE 8:** Association of average FPG with other variables.

Name of variables	Fasting plasma glucose			Chi‐square (*p*)
	Normal (< 8)	Moderate (8–12)	High (> 12)	
Gender				0.722 (0.697)
Male	75 (156.3)	106 (46.5)	5 (8.6)	
Female	60 (125)	86 (37.7)	2 (3.4)	
Treatment type				2.431 (0.657)
Only insulin	0 (0)	1 (0.4)	0 (0)	
Only OAD	33 (68.8)	36 (15.8)	1 (1.7)	
Insulin and OAD	102 (212.5)	155 (68)	6 (10.3)	
Follow doctors' advice				24.531 (0.000)
Take medicine	33 (68.8)	83 (36.4)	6 (10.3)	
Regular exercise	14 (29.2)	28 (12.3)	0 (0)	
Take medicine and regular exercise	88 (183.3)	81 (35.5)	1 (1.7)	
Maintained food habit				2.070 (0.355)
Yes	42 (87.5)	46 (20.2)	2 (3.4)	
No	93 (193.8)	146 (64)	5 (8.6)	
Do exercise				10.090 (0.006)
Yes	34 (70.8)	23 (10.1)	2 (3.4)	
No	101 (210.4)	169 (74.1)	5 (8.6)	
BMI				8.023 (0.091)
18.5 < BMI < 25	63 (131.3)	91 (39.9)	3 (5.2)	
25 < BMI < 30	38 (79.2)	74 (32.5)	3 (5.2)	
BMI > 30	34 (70.8)	27 (11.8)	1 (1.7)	

### 3.1 Statistical Modelling

**FIGURE 5:**
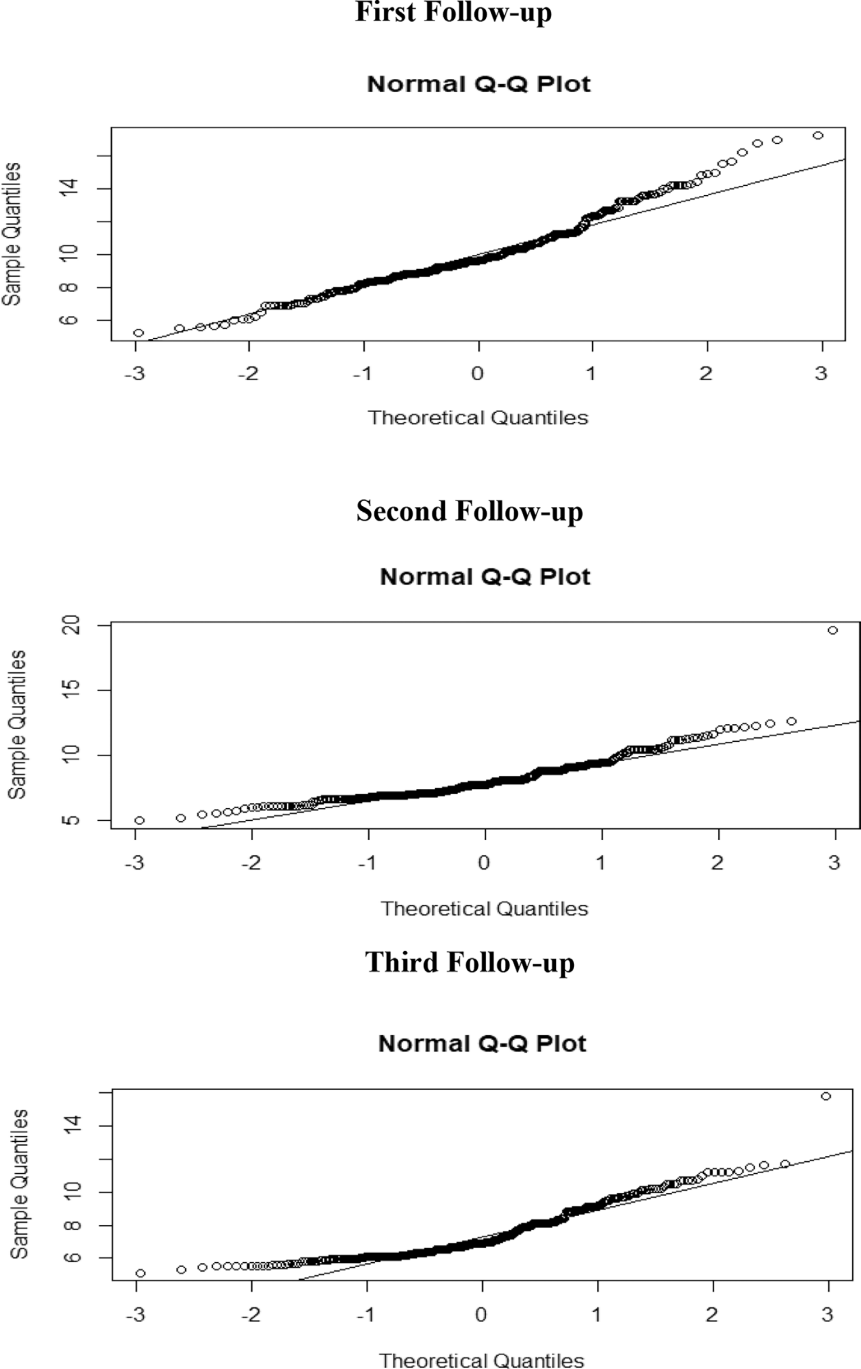
Q‐Q plot for TESTING normality of the fasting plasma glucose.

The Q‐Q plots reveal significant deviations from normality in the Fasting Plasma Glucose data across all follow‐ups (Figure 5), which is confirmed by the Shapiro–Wilk test results presented in Table 9. The test statistics (0.95873, 0.9057 and 0.8631) for the first, second and third follow‐ups, respectively, along with extremely small *p*‐values (4.292 × 10^−08^, 3.912 × 10^−07^ and 3.122 × 10^−07^), indicate consistent violations of the normality assumption at a 5% significance level (*α* = 0.05) throughout the study period.

**TABLE 9:** Shapiro–Wilk test statistic and *p*‐value for three follow‐ups.

Follow‐up	Test statistic	*p*	Decision
Follow‐up one	0.95873	$4.292\times 10^{-08}$	Null rejected
Follow‐up two	0.9057	$3.912\times 10^{-07}$	Null rejected
Follow‐up three	0.8631	$3.122\times 10^{-07}$	Null rejected

### 3.2 Logistic Regression Model

Table 10 presents logistic regression results showing that for all three follow‐ups, the test statistics (13.263, 12.463 and 14.643) correspond to *p*‐values (0.06594, 0.08594 and 0.05594), which are above the 5% significance level (*α* = 0.05). These findings indicate that the null hypothesis is accepted, suggesting no violation of the proportional odds assumption across the three follow‐up periods.

**TABLE 10:** The Brant test statistic and *p*‐value for three follow‐up periods.

Follow‐up	Test statistic	*p*	Decision
Follow‐up one	13.263	0.06594	Null accepted
Follow‐up two	12.463	0.08594	Null accepted
Follow‐up three	14.643	0.05594	Null accepted

### 3.3 Follow‐Up

In the first follow‐up, individuals in the moderate threshold category had a significant increase in the odds of the outcome (odds ratio = 10.359, *p* = 0.036) compared to those in the normal category, though the difference between moderate and high thresholds was not significant. Females had significantly lower odds (odds ratio = 0.409, *p* = 0.001) compared to males and the group (case vs. control) significantly influenced diabetes mellitus (*p* < 0.05), while exercise, co‐morbidity, time duration, rest sleep time, bad habits and BMI did not significantly influence the outcome (*p* > 0.05). In the second follow‐up, co‐morbidity, rest sleep time, bad habits and BMI were found to be insignificant, while group, gender, exercise and time duration emerged as significant predictors. The moderate‐high threshold category showed a substantial increase in the odds of the outcome (odds ratio = 47.967, *p* < 0.0001) and the control group demonstrated significantly lower odds (odds ratio = 0.078, *p* = 0.001) compared to the case group. In the third follow‐up, females exhibited higher odds (odds ratio = 4.329, *p* = 0.03) compared to males and individuals who did not exercise regularly had higher odds (odds ratio = 1.202, *p* < 0.0001) compared to those who did. Variables like co‐morbidity, rest sleep time and BMI did not show significant effects on the outcome (*p* > 0.05) (Tables 11, 12, 13).

**TABLE 11:** Proportional odds logistic regression model for fasting plasma glucose based on estimated effects of selected covariates.

Variables	Characteristics	Estimate	Odds ratio	Std. error	*z*	Pr (> *z*)
Threshold	Normal | Moderate	−1.185	0.306	1.109	−1.069	0.285
	Moderate | High	2.338	10.359	1.115	2.097	0.036
Group	Control	Reference	—	—	—	—
	Case	0.014	1.066	0.234	0.274	0.784
Gender	Male	Reference	—	—	—	—
	Female	−0.895	0.409	0.278	−3.223	0.001
Exercise	Yes	Reference	—	—	—	—
	No	0.272	1.313	0.312	0.874	0.382
Co‐morbidity	Yes	Reference	—	—	—	—
	No	−0.065	0.937	0.252	−0.259	0.796
Time duration	Continuous	−0.046	0.955	0.169	−0.274	0.784
Rest sleep time	Continuous	0.136	1.145	0.126	1.076	0.282
Bad habit	Yes	Reference	—	—	—	—
	No	−0.094	0.910	0.326	−0.289	0.773
BMI	Continuous	−0.057	0.944	0.0516	−1.107	0.268

**TABLE 12:** Proportional odds logistic regression model for postprandial plasma glucose based on estimated effects of selected covariates.

Variables	Characteristics	Estimate	Odds ratio	Std. error	*z*	Pr (> *z*)
Threshold	Nominal | Moderate	−0.630	0.532	1.256	−0.502	0.616
	Moderate | High	3.871	47.967	1.309	2.957	0.003
Group	Control	Reference	1	—	—	—
	Case	−2.887	0.055	0.300	−9.633	0.000
Gender	Male	Reference	1	—	—	
	Female	0.688	1.989	0.309	2.224	0.026
Exercise	Yes	Reference	1	—	—	—
	No	0.301	1.351	0.363	4.830	0.007
Co‐morbidity	Yes	Reference	1	—	—	
	No	0.121	1.128	0.289	0.417	0.676
Time duration	Continuous	−0.559	0.571	0.197	−2.837	0.005
Rest sleep time	Continuous	0.113	1.119	0.142	0.795	0.426
Bad habit	Yes	Reference	1	—	—	—
	No	0.304	1.355	0.387	0.786	0.432
BMI	Continuous	−0.073	0.928	0.0450	−1.635	0.102

**TABLE 13:** Proportional odds logistic regression model for HbA1c based on estimated effects of selected covariates.

Variables	Characteristics	Estimate	Odds ratio	Std. error	*z*	Pr (> *z*)
Threshold	Nominal | Moderate	2.501	0.532	1.57	1.588	0.814
	Moderate | High	8.681	47.967	1.932	4.493	0.0001
Group	Case	Reference	1	—	—	—
	Control	−2.8524	0.078	0.034	4.40	0.001
Gender	Male	Reference	1	—	—	—
	Female	1.465	4.329	40.77	2.224	0.03
Exercise	Yes	Reference	1	—	—	—
	No	0.184	1.202	48.02	0.830	0.000
Co‐morbidity	Yes	Reference	1	—	—	—
	No	0.1812	1.198	35.66	0.417	0.504
Time duration	Continuous	0.1583	1.171	24.39	−2.837	0.04
Rest sleep time	Continuous	0.283	1.327	18.18	0.795	0.403
Bad habit	Yes	Reference	1	—	—	—
	No	0.1536	1.166	46.86	5.786	0.001
BMI	Continuous	−0.007825	0.9922	47.3	0.840	0.500

## 4 Discussion

This study demonstrates that 20% of the case group and 94% of the control group participants did not engage in regular exercise. Furthermore, 10% of the control group and 7% of the case group failed to maintain a proper diet. Physical activity enhances insulin sensitivity, promotes glucose uptake by skeletal muscles and improves overall metabolic health [12]. Conversely, unhealthy dietary habits, such as high consumption of refined carbohydrates, saturated fats and sugary beverages, are strongly linked to an increased risk of type 2 diabetes mellitus (T2DM) [13]. A recent systematic review revealed that the risk of type 2 diabetes decreased significantly at a walking speed of 4 km/h and above [14]. A prospective cohort study stated that adherence to a healthy lifestyle at mid‐life is associated with a longer life expectancy free of major chronic diseases like diabetes, cardiovascular disease and cancer [15]. The World Health Organization (WHO) and the International Diabetes Federation emphasise the importance of lifestyle interventions, including culturally tailored diet and physical activity programs, to effectively prevent and manage T2DM [16, 17]. Regular exercise also reduces the risk of cardiovascular diseases, which are common comorbidities in T2DM patients [4]. In this study, 44% of respondents had comorbidities, with hypertension being the most prevalent, illustrating the complexity of health conditions in diabetic patients.

The structured physical activity and dietary modifications led to significant improvements in FPG, PPG and HbA1c levels, supporting previous study reports regarding the critical role of structured lifestyle intervention programs in diabetes management [18]. The study's findings can guide healthcare practitioners in creating personalised treatment plans that integrate lifestyle modifications, thereby improving diabetes management and patient quality of life.

A significant proportion of study participants were either overweight or obese both in the case and control groups, emphasising the need for effective weight management strategies in diabetes prevention and treatment. The overall RR of developing Type 2 diabetes for obese and overweight persons compared with normal weight persons was 7.9 and 2.99 times higher respectively [19]. Obesity is characterised by an increased inflammatory status, being a metabolic risk factor [20] while excess body weight, especially central adiposity, exacerbates insulin resistance and increases T2DM risk [21]. Appropriate dietary patterns and the right intake of Mg^2+^ improve metabolic syndrome by reducing blood pressure, hyperglycaemia and hypertriglyceridemia [21]. Public health efforts should therefore prioritise weight management strategies that combine physical activity, nutritional guidance, smoking cessation and reduced alcohol consumption. Urban environments should also be designed to encourage physical activity by providing pedestrian‐friendly infrastructure and accessible recreational spaces [22].

The distribution of gender, education levels and professions among individuals with T2DM in this study is consistent with previous research highlighting the role of socioeconomic status in diabetes prevalence and management [23]. The predominance of individuals in the medium‐income range suggests that socioeconomic status may influence health outcomes. This underscores the importance of personalised interventions that consider demographic characteristics, ensuring that public health strategies are tailored to the needs of diverse populations.

Adherence to medical advice was a significant factor in glycaemic control in this study, with 92% of participants following healthcare recommendations. This finding aligns with current clinical guidelines, which advocate for personalised treatment plans that emphasise lifestyle modifications alongside pharmacological interventions [2]. By following medical recommendations and adopting appropriate treatment modalities, patients can better manage glucose levels and reduce complication risks.

These findings suggest that public health interventions should address the multifaceted nature of T2DM. Targeted efforts to improve health literacy, promote healthy behaviours and enhance access to healthcare services may help reduce the T2DM burden. Addressing modifiable risk factors and considering socioeconomic determinants can improve overall public health outcomes.

A strong positive correlation between FPG and postprandial plasma glucose (PPG) across all follow‐ups and the substantial positive correlation between glycated haemoglobin (HbA1C%) and PPG highlights the influence of long‐term glycaemic control on postprandial glucose levels. The weak negative correlation between HbA1C% and haemoglobin suggests a nuanced relationship between long‐term glycaemic control and haemoglobin levels. These insights can inform clinical decision‐making, guide interventions and contribute to a holistic approach to diabetes management. Persistent gender disparities in FPG levels emphasise the need to address social determinants of health in diabetes care. Significant associations between FPG levels, medical advice and treatment type underscore the critical role of healthcare professionals in diabetes management [24, 25]. This study with follow‐ups at 3 and 6 months evaluated the effects of structured lifestyle modifications.

This study has several limitations, including its cross‐sectional design, which limits the ability to establish causal relationships. The duration of diabetes was recorded but not matched between groups, potentially influencing glycaemic outcomes. Future studies should consider stratified analysis by diabetes duration and employ longitudinal designs to assess the sustained effects of lifestyle modifications. Additionally, self‐reported adherence to physical activity and dietary changes may introduce recall bias; objective measures such as accelerometers and validated dietary assessments should be used in future research. Genetic predisposition, environmental influences and psychosocial factors were not explored, which may impact type 2 diabetes mellitus (T2DM) management. The study was conducted at a single hospital, limiting generalisability. Future multi‐centre studies should validate these findings and explore cost‐effective, scalable interventions. Targeted interventions should address modifiable risk factors to mitigate their long‐term impact on glycaemic control and diabetes‐related complications.

## 5 Conclusion

Our findings indicate that case group patients demonstrated substantial improvements in diabetes management when compared with control group patients, including a significant decrease in Fasting Plasma Glucose (FPG) levels across two follow‐ups after the baseline assessment, reflecting enhanced glycaemic control. The analysis underscores the critical role of lifestyle factors—such as exercise and diet—in influencing FPG levels. The interventions have effectively promoted healthier habits, such as engaging in at least 150 min of weekly physical activity and adhering to a low‐sugar, high‐fibre diet for improving diabetes outcomes.

## Author Contributions

Hasan Mahmud Hadi: conceptualization, data curation, formal analysis, investigation, methodology, visualization, writing – original draft. Md. Monir Hossain Shimul: methodology, Validation, visualization, writing – original draft, and writing – review and editing. Md. Sakhawath Hossain: data collection, follow‐up and field supervision. Afroza Sultana: formal analysis, visualization. Md. Kamrul Hossain: statistical analysis, visualization. Salamat Khandker: methodology, supervision, writing – review and editing. Salim Khan: methodology, writing – review and editing.

## Conflicts of Interest

The authors declare no conflicts of interest.

## Data Availability

The data supporting the conclusions of this article are included within the article.
